# Improved Anti-Vulcanization and Bonding Performance of a Silver Alloy Bonding Wire by a Cathodic Passivation Treatment with Palladium

**DOI:** 10.3390/ma15072355

**Published:** 2022-03-22

**Authors:** Guannan Yang, Zhiqiang Zhou, Haide Zhang, Yu Zhang, Zhen Peng, Pan Gong, Xin Wang, Chengqiang Cui

**Affiliations:** 1 State Key Laboratory of Precision Electronic Manufacturing Technology and Equipment, Guangdong University of Technology, Guangzhou 510006, China; ygn@gdut.edu.cn (G.Y.); 2111901233@mail2.gdut.edu.cn (Z.Z.); 3119000357@mail2.gdut.edu.cn (H.Z.); 2 Jihua Laboratory, Foshan 528225, China; 3 School of Materials Science and Engineering, Jiangsu University, Zhenjiang 212013, China; 4 State Key Laboratory of Materials Processing and Die & Mould Technology, School of Materials Science and Engineering, Huazhong University of Science and Technology, Wuhan 430074, China; pangong@hust.edu.cn; 5 Key Laboratory for New Type of Functional Materials in Hebei Province, School of Materials Science, Hebei University of Technology, Tianjin 300401, China; ahaxin@hebut.edu.cn

**Keywords:** silver alloy bonding wire, bonding strength, vulcanization resistance, cathodic passivation

## Abstract

As a traditional interconnect material, silver alloy bonding wires are widely used in electronic packaging, but their propensity to vulcanize quickly has not been sufficiently addressed. The current surface anti-oxidation and anti-sulfidation treatments are often accompanied by a decline in bonding performance, which hinders the use of silver alloy bonding wires in new applications. In the present paper, we develop a new cathodic passivation treatment in a Pd-containing solution for silver bonding wires, which not only significantly improves their vulcanization resistance, but also maintains their bonding performance. The surface of the treated wires remains unaffected after vulcanization in 0.3 μg/m^3^ of ammonium sulfide for 60 min. Compared to a Pd-free passivation treatment, the bonding strength of the wire passivated with the Pd-containing solution improves from 0.20 to 0.27 N. XPS analysis confirms the existence of Pd on the surface of the wire. The solder ball formed an obtuse angle instead of a sharp angle on the pad, which is beneficial for bonding strength.

## 1. Introduction

As a traditional packaging interconnect technology, wire bonding still occupies an important place in electronic packaging, due to its simple process and low cost [1]. The materials for bonding wires have developed from pure metals to a variety of alloys based on, for example, gold, silver, copper, and aluminum [2,3,4,5]. Among these metals, silver has the best electrical and thermal conductivity, and its price is relatively moderate. Therefore, silver bonding wires have been considered as a potential alternative to gold bonding wires. However, silver alloy bonding wires can be easily contaminated when used in an environment without a protective gas [6,7], which becomes an obstacle for extending their use to new applications.

Bonding reliability is another important property of bonding wires. The device will fail, as long as one of the bonding points is damaged. Generally, the failure modes of the solder ball of wire bonding can be divided into intra-ball failure and interfacial failure, which usually start from the intermetallic compounds, interfacial voids and interfacial corrosion points [8,9]. Zhang et al. [10] studied the failure of LED packages and pointed out that the stress concentration generated in the thermal shock test can lead to the neck fracture of the solder balls. On the other hand, interfacial failures are also widely observed. Wu et al. [11] conducted power cycle life tests on different types of insulated gate bipolar translator (IGBT) modules, and found that the recrystallization and electrothermal migration could lead to the fracture of the bonding interface. Zheng et al. [12] found that the separation of the bonding wire and the pad is the main failure mode of IGBT modules. The mismatch of thermal expansion coefficients between the bonding wire and the chip causes stress concentration and leads to cracks at the bonding interface to form an open circuit.

To address these problems, various technologies, such as alloying and surface treatments, were developed. For instance, Cheng and Hsiao [13] tried to improve the oxidation and interfacial corrosion resistance of Ag wires through the addition of Au and Pd. Guo and Jong-Soo [14] revealed that doping with Pd can improve the bonding force of the wire by increasing the interconnect reliability between the solder ball and the pad interface. Tseng et al. [15] plated silver wires with gold to improve its oxidation resistance and mechanical properties. Fei-Yi et al. [16] galvanized aluminum bonding wires to control the shape of the solder ball. However, new problems appeared with these treatments. After the bonding wire was alloyed, its electrical and thermal conductivity decreased, which would lead to an increase in manufacturing costs [17]. The thickness of the noble metal element electroplated on the bonding wires was relatively large (~100 nm) and also expensive [18]. The application of a surface treatment affected the mechanical performance of the bonding wires, thereby reducing the life of the package structure [16,19]. As a result, finding a new anti-vulcanization processing method that combines low cost, facile operation, and minimal influence on the bonding performance remains a challenge for the use of silver bonding wires.

In this study, we develop a new cathodic passivation solution for the anti-vulcanization treatment of silver bonding wires. Through the addition of palladium nitrate in solution, the vulcanization resistance of the passivated wires is significantly improved. On the other hand, the treatment also improved the surface wettability of the bonding wires on Au pads. Therefore, the solder ball tends to form an obtuse angle instead of a sharp angle on the pad, which is beneficial to reducing the interface stress concentration and improving the bonding strength. This study could provide a way to improve the sulfidation resistance of bonding wires with less impact on the bonding performance, and shows that wettability modulation may be an effective way to improve the reliability of bonding interfaces.

## 2. Experiments

### 2.1. Preparation of the Materials 

Silver alloy bonding wires with a diameter of 20 μm were purchased from Xinqipai Electronic Technology Co., Ltd., Chongqing, China. The compositions of the silver alloy bonding wires are shown in Table 1. A hydrotropic solution with 0.010 mol/L of chromium acetate, 0.008 mol/L of trisodium citrate, 0.010 mol/L of sodium tartrate, 0.015 mol/L of crystalline sodium acetate, and 0.140 mol/L of sodium hydroxide was used as the Pd-free passivation solution. Another Pd-containing passivation solution was prepared by adding 0.001 mol/L of palladium nitrate to the Pd-free passivation solution.

### 2.2. Experimental Methods

**Figure 1:**
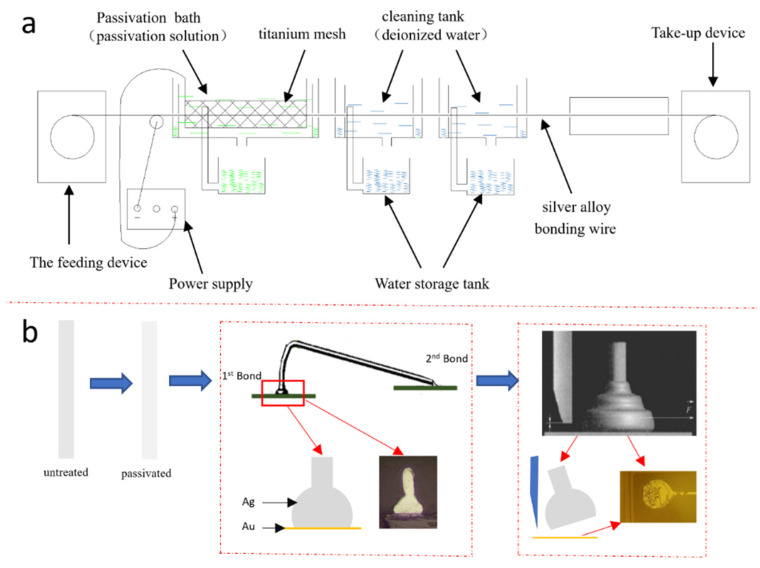
(**a**) Illustration of the cathodic passivation device. (**b**) Illustrations of the wire bond and the shear force test.

Figure 1a provides an illustration of the cathodic passivation device. The silver alloy bonding wire was sequentially immersed into the passivation solution and deionized water. A current of 500 A/m^2^ was applied to the wires and the passivation solution. The wire is the cathode. The wire was processed in the passivation solution for 5 s at 25 °C.

Figure 1b shows a flow chart of the experimental procedures after the passivation treatment. The original silver wires and the passivated wires in the Pd-free and Pd-containing solutions were bonded onto Au pads (diameter = 50 μm) using an automatic bonding tool (AW386, Ada Intelligent Equipment Co., Ltd., Foshan, China) for the ball shear force test. The wires formed two bond points, the first being the ball bond and the second being the wedge bond. The detailed bonding parameters are shown in Table 2. After the bonding wires were attached on the Au pads, the cross-sections of the ball bonds on the Au pads were observed by a scanning electron microscope (SEM, HITACHI SU8220, Tokyo, Japan). Using a thrust tester (Condor Sigma, XYZTEC), the ball bonds were pushed away from the Au pads, and the bonding strength was recorded. The fracture morphologies of the Au pads after the shear tests were observed by the SEM.

The original silver wires and the passivated wires in the Pd-free and Pd-containing solutions were placed in a sealed 3 × 3 × 3 cm^3^ box filled with ammonium sulfide gas with a concentration of 0.3 μg/m^3^. The surface condition of the wires was recorded by an optical microscope. Due to the small size of the silver alloy bonding wires, silver plates with a thickness of 10 μm were passivated in the solutions using the same parameters. The surfaces of the passivated silver plates were analyzed by X-ray photoelectron spectroscopy (XPS, Escalab 250Xi).

## 3. Results and Discussions

### 3.1. Vulcanization Resistance of the Passivated Silver Alloy Bonding Wires in Different Solutions

**Figure 2:**
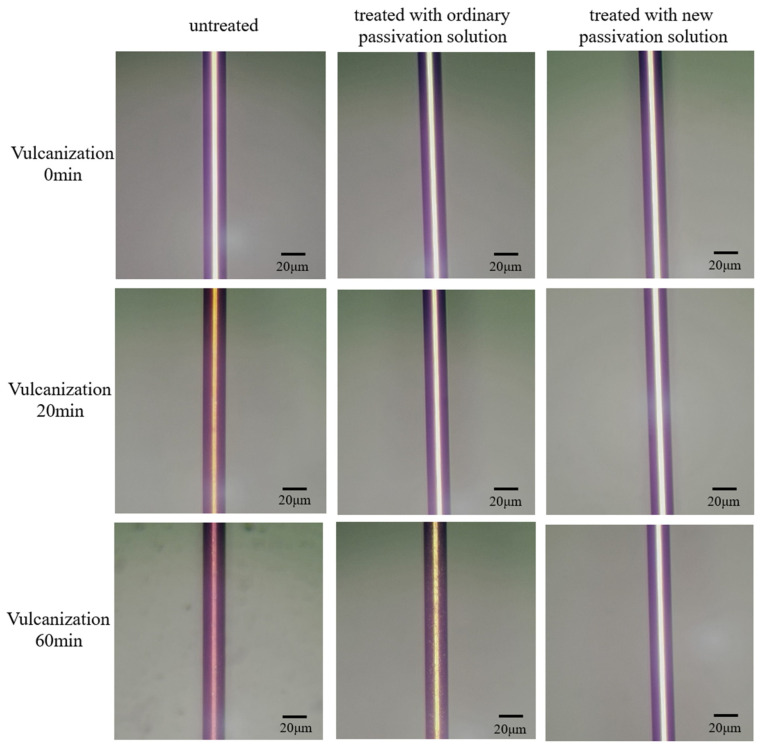
Comparison of the surface of the passivated silver alloy bonding wires in different solutions, before and after vulcanization for 20 and 60 min.

Figure 2 shows the surface of the passivated silver alloy bonding wires in different solutions, before and after vulcanization in ammonium sulfide for 20 and 60 min. The surface of the untreated silver alloy bonding wire turned yellow after 20 min of vulcanization, and the surface was further vulcanized to dark red after 60 min. The surface of the silver alloy bonding wire treated with the standard, Pd-free passivation solution turned pale yellow after vulcanization for 20 min. After 60 min of vulcanization, the surface of the bonding wire darkened further. The surface of the silver alloy bonding wire treated with the new Pd-containing passivation solution after vulcanization for 60 min had the same appearance and silver-white color as the surface of the wire before vulcanization. This result indicate that the Pd-containing passivation solution provides better vulcanization resistance than the Pd-free passivation solution.

### 3.2. Surface Analysis

**Figure 3:**
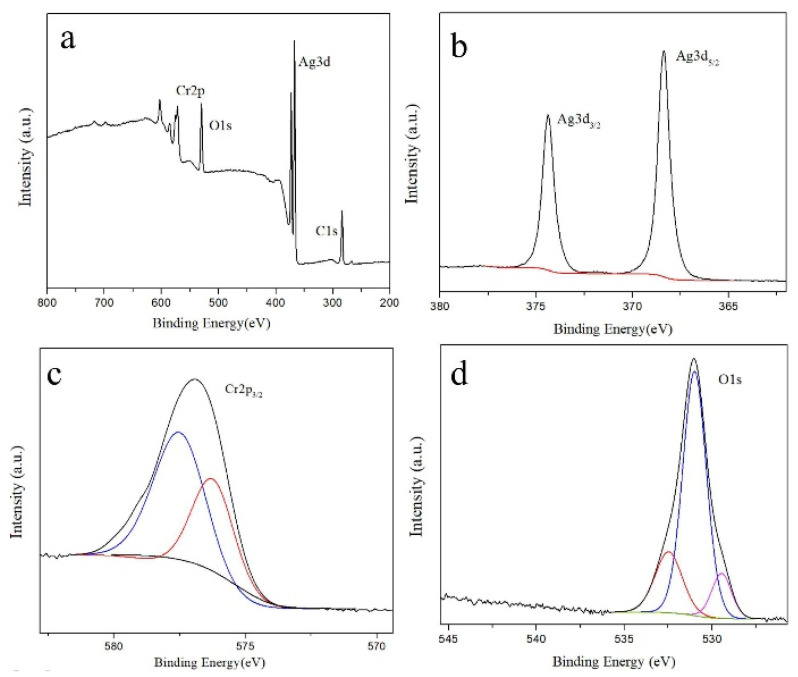
XPS spectra of the passivated silver plates in the Pd-free solution after storage for 5 days. (**a**) Survey spectra; (**b**) Ag3d spectra; (**c**) Cr2p spectra; (**d**) O1s spectra.

**Figure 4:**
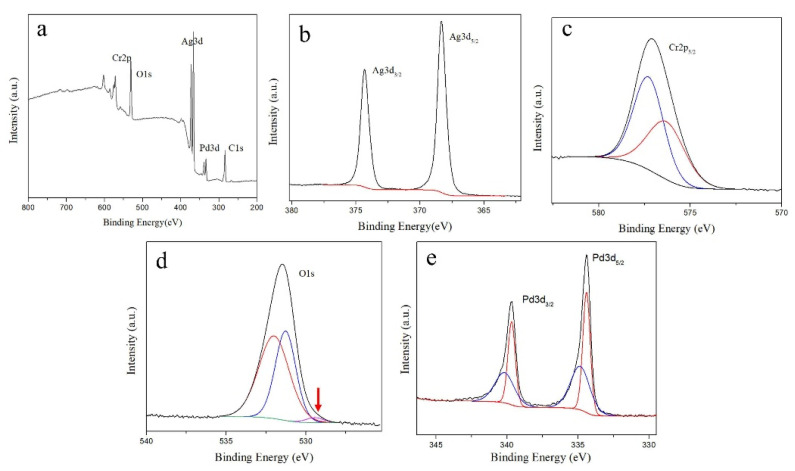
XPS spectra of the passivated silver plates in the Pd-containing solution after storage for 5 days. (**a**) Survey spectra; (**b**) Ag3d spectra; (**c**) Cr2p spectra; (**d**) O1s spectra; (**e**) Pd3d spectra.

Figure 3 shows the XPS spectra of the passivated silver plates in the Pd-free solution after storage for 5 days. Peaks of Ag3d, O1s, Cr2p, and C1s electrons are apparent. The Ag3d fine spectrum (Figure 3b) shows 2 characteristic peaks at binding energies of 368.40 and 374.40 eV, corresponding to the Ag3d_5/2_ and Ag3d_3/2_ electrons, respectively. It has been reported that the peaks of Ag are at 368.2 eV [20] and 374.27 eV [21], and the peaks of Ag_2_O are at 367.4 eV [22] and 373.90 eV [23], respectively. As the peaks of the Ag3d fine spectrum in Figure 3b show highly symmetrical shapes, it can be concluded that most of the Ag element is in the form of Ag, but not AgO. The Cr2p_3/2_ fine spectrum (Figure 3c) shows 2 characteristic peaks at binding energies of 577.28 and 576.30 eV, corresponding to Cr(OH)_3_ and Cr_2_O_3_, respectively [24,25,26], The peak of Cr is at 574.13 eV [27], which is not observed in the spectrum in Figure 3. The Cr ions are present because they are in the passivation solution. The O1s fine spectrum (Figure 4d) shows 3 characteristic peaks after peak fitting, with binding energies of 532.00, 531.28 and 529.44 eV, respectively. The peak at 529.44 eV corresponds to O^2−^ [28], which indicates that the surface contains metallic oxides. The peak at 531.28 eV corresponds to organic C–O and OH^−^ [29,30], and the peak at 532.00 eV corresponds to organic C=O and OH^−^ [29,31]. These two peaks and the existence of the C1s peak indicate that the surface of the passivated silver plate contains some residual organics, which are difficult to avoid in XPS measurements [32]. Based on the XPS spectra, it can be concluded the metallic species on the surface of the passivated silver plates in the Pd-free solution are Ag, Cr(OH)_3_ and Cr_2_O_3_.

Figure 4 shows the XPS spectra of the passivated silver plates in the Pd-containing solution after storage for 5 days. Peaks of Ag3d, O1s, Pd3d, Cr2p, and C1s electrons can be observed. The spectra of Ag3d (Figure 4b) and Cr2p (Figure 4c) are similar to that of the passivated silver plates in the Pd-free solution. In the O1s fine spectrum (Figure 4d), the O^2-^ peak at 529.44 eV is relatively smaller than that in Figure 3d, as marked by the red arrow. In Figure 4e, the Pd3d_3/2_ fine spectrum shows 2 characteristic peaks at 340.18 and 339.68 eV, and the Pd3d_5/2_ fine spectrum shows 2 characteristic peaks at 334.85 and 334.41 eV. These peaks correspond to Pd [33]. The peaks of PdO, PdO_2_ and PdO_3_ are at 336.90, 337.50 and 337.7 eV, respectively [34,35], which are not observed in the spectrum in Figure 4. Their absence indicates that the Pd on the surface of the passivated silver plate is in the form of Pd. Based on the XPS spectra, it can be concluded the metallic species on the surface of the passivated silver plates in the Pd-containing solution mainly contains Ag, Cr(OH)_3_, Cr_2_O_3_ and Pd. Based on the XPS spectra, the relative atomic concentrations of different metallic species on the surfaces of the passivated Ag plates are calculated and shown in Table 3. In comparison, the surface of the silver plates in the Pd-containing solution contains a small amount of Pd, and the concentrations of Ag and Cr_2_O_3_ have slightly decreased.

### 3.3. Shear Tests of the Passivated Silver Alloy Bonding Wires in Different Solutions

**Figure 5:**
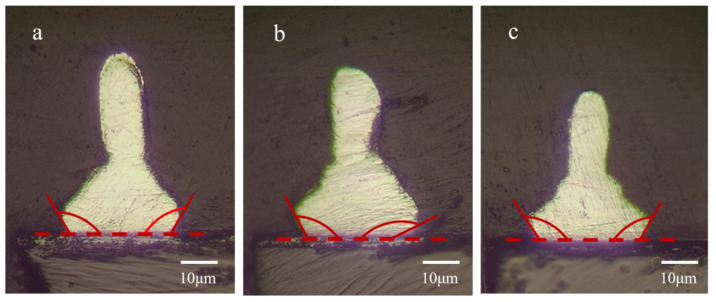
Cross-section images of the bonded wires on the pads before the shear force tests. (**a**) Untreated wire. (**b**) Wire passivated in the Pd-free solution. (**c**) Wire passivated in the Pd-containing solution.

Figure 5 shows the cross-section images of the bonded wires on the pads before the shear force tests. As the wires are melted during the wire bonding process, the solder ball and the pad form a contact angle, which are summarized in Table 4. For the untreated wire (Figure 5a), the average contact angle is 104°, whereas for the wires passivated in the Pd-free solution (Figure 5b), the average contact angle is 136°, corresponding to a lower wettability. As a result, the contact interface between the solder ball and the pad tends to shrink, and eventually forms a sharp angle on the edge. Due to the stress concentration caused by the sharp angle and the relatively low contact area, a low bonding strength can be expected. For the wire passivated in the Pd-containing solution (Figure 5c), the average contact angle decreased to 108°, which is close to the contact angle of the untreated wires.

One possible reason for the different contact angles of the wires relates to the content of elements on the surface of the wires. For the wires passivated in the Pd-containing solution, there is a small amount of Pd on the surface. As revealed in previous studies [36,37,38,39,40], the existence of noble metal elements, such as Pd, is beneficial for the oxidation and vulcanization resistance of the Cu and Ag bonding wires. The presence of Pd in the passivated layer might hinder the oxidation of the wires during the bonding process, which decreases the surface energy and increases the wettability of the solder ball. Therefore, smaller contact angles are formed.

**Figure 6:**
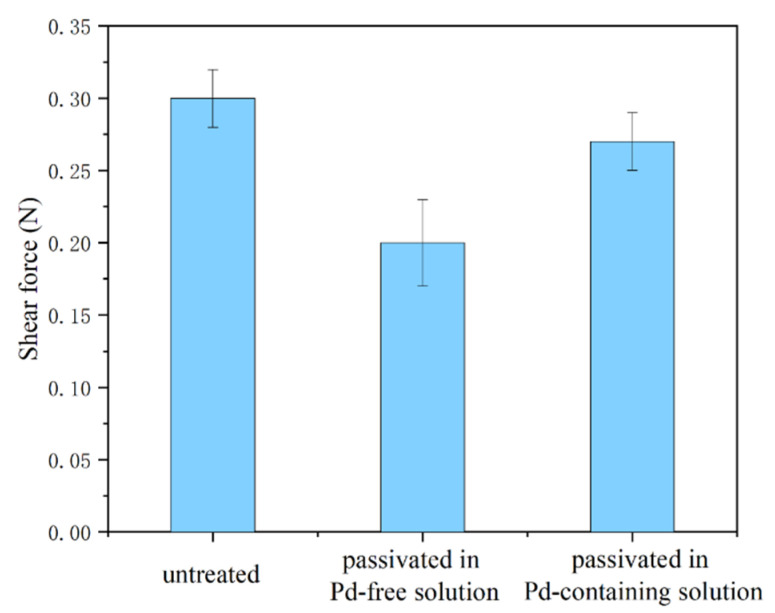
Bonding strength of the untreated silver alloy bonding wires and the silver alloy bonding wires passivated in different solutions.

Figure 6 shows the measured bonding strength of the wires. The untreated wires, the wires passivated in the Pd-free solution, and the wires passivated in the Pd-containing solution. fractured at mean shear forces of 0.30, 0.20, and 0.27 N, respectively. Through the use of the Pd-containing solution for passivation, the strength of the treated bonding wire increased by 35%, compared to the wire treated in the standard Pd-free solution.

**Figure 7:**
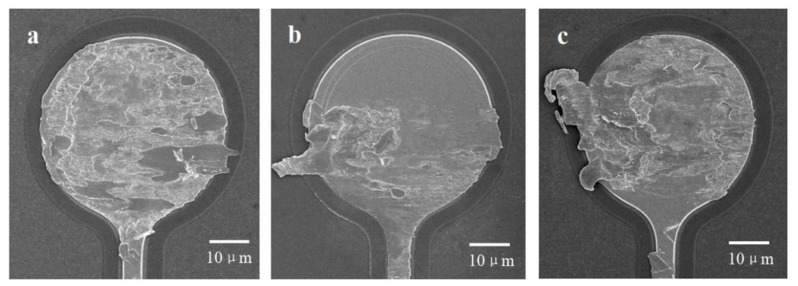
Fracture surface of the untreated and passivated silver alloy bonding wires in different solutions. (**a**) Untreated wire. (**b**) Wire passivated in the Pd-free solution. (**c**) Wire passivated in the Pd-containing solution.

Figure 7 shows the fracture surface of the untreated silver alloy bonding wires and silver alloy bonding wires passivated in different solutions. For the untreated wires, the whole fracture surface is covered by striped fracture patterns, indicating a good interconnection between the bonding wire and the pad. The average bond force is relatively high (0.30 N). For the passivated wire in the Pd-free solution, only half of the fracture surface is covered with the fracture pattern, and the other half of the surface retains the original flat morphology. The presence of some of the original morphology indicates that the bonding wire has not made sufficient contact with the pad. The average bond force is relatively low (0.20 N). For the wire passivated in the Pd-containing solution, the fracture surface is almost completely covered with the fracture pattern, indicating a good interconnection between the bonding wire and the pad. The average bond force is close to that of the untreated wires (0.27 N).

Based on the measured contact angles and bonding strength, it can be found that a smaller contact angle corresponds to a higher strength. This is because the obtuse angle between the solder ball and the pad is beneficial to reducing the stress concentration on the interface. These results indicate that wettability modulation may be an effective way to improve the interface reliability of wire bonding.

Based on the above results and discussion, the improved vulcanization resistance and bonding performance of the silver alloy bonding wires passivated in the Pd-containing solution can be reasonably understood. The results show that Pd plays a key role in improving the performance of the bonding wire. If Pd is added to the bonding wire by alloying, the required amount of Pd is relatively high, which adds to product cost and may lead to problematic inhomogeneous compositions. In comparison, the cathodic passivation method has the advantages of simple processing, fast reaction times, reduced material consumption, and applicability for different material components.

## 4. Conclusions

A cathodic passivation solution with palladium nitrate has been developed to improve the resistance to vulcanization while retaining the bonding performance of silver alloy bonding wires. For the passivated wires treated with a Pd-free solution, the vulcanization resistance is improved, but the bonding strength of the wire significantly decreases from 0.30 to 0.20 N. With the addition of palladium nitrate to the passivation solution, the surface of the wire can maintain its original silver-white color after vulcanization in 0.3 μg/m^3^ of ammonium sulfide for 60 min. The wettability of the wires is improved, as the contact angle between the solder balls and the Au pad decreases from 136° to 108°. The bonding strength of the wire is 0.27 N, 35% stronger than the wire treated with the Pd-free solution. The cathodic passivation treatment in the Pd-containing solution in this study provides an effective and facile method for the improvement of the properties of silver alloy bonding wires.

## Figures and Tables

**Table 1:** Compositions of the silver alloy bonding wires.

Element	Ag (%)	Fe (ppm)	Pd (ppm)	Mg (ppm)	Si (ppm)
Content	≥99	≤5	≤1	≤2	≤1

**Table 2:** Bonding parameters for the silver bonding wires.

Parameters	1st Bond	2nd Bond
Bonding time (ms)	14	14
Power (mW)	55	80
Pressure (gf)	24	40
Bonding temperature (°C)	150	150
Electronic flame-off current (mA)	28	0
Electronic flame-off time (μs)	530	0

**Table 3:** Relative atomic concentrations of different metallic species on the surfaces of the passivated Ag plates.

Treatment	Concentrations (in at. %)			
	Ag	Cr(OH)_3_	Cr_2_O_3_	Pd
Passivated in Pd-free solution	68.8%	16.4%	9.2%	-
Passivated in Pd-containing solution	62.3%	13.5%	8.4%	15.8%

**Table 4:** Measured contact angles of the bonded wires on the pads.

Wire Treatment Type	Contact Angle (°)			
	Maximum	Minimum	Mean	Standard Error
Untreated	113	93	104	8
Passivated in Pd-free solution	157	104	136	16
Passivated in Pd-containing solution	120	94	108	8

## Data Availability

The datasets generated during and/or analyzed during the current study are available from the corresponding author on reasonable request.
